# Commerce of Edible Insects in the State of Morelos, Mexico

**DOI:** 10.1093/jisesa/ieaa106

**Published:** 2020-10-16

**Authors:** José Manuel Pino Moreno, Humberto Reyes-Prado

**Affiliations:** 1 Zoology Department, Entomology Laboratory, Biology Institute, Universidad Nacional Autonoma de Mexico, CDMX, México; 2 Chemical Ecology Laboratory, ESS Jicarero-Universidad Autonoma del Estado de Morelos. Jojutla de Juárez, Morelos, México

**Keywords:** anthropo-entomophagy, trade, grasshoppers, jumiles’

## Abstract

The present study deals with the taxonomical analysis of the edible insects commercialized in the state of Morelos, Mexico. We have recorded two species under the order Orthoptera and four species under Hemiptera–Heteroptera. Our work revealed that grasshoppers and small hemipterans known as ‘jumiles’ are the two main insects sold in the markets of the state of Morelos. We also discuss insects’ prices and their economic importance for the livelihood improvement of the local people connected to the edible insect trade.

Anthropo-entomophagy is the consumption of insects or insect-based products by humans (van Huis 2015). Despite the abundant insect resources that ecosystems provide to society, malnutrition continues to prevail in Latin America and the Caribbean. Malnourishment is the most important factor contributing to child mortality in developing countries (Guardiola and Gónzalez-Gómez 2010).

Socioeconomic differences are conspicuous in Latin America: problems of marginalization, lack of access to mass media, and educational deficiencies are faced daily. The evident problems of hunger and malnutrition in Latin America have been noted by several authors and institutions and identified as priority issues (Guardiola and Gónzalez-Gómez 2010). A low nutrient intake has been one of the main long-standing reasons why native people eat numerous insect species since they provide nutrients that basic foods lack (Meyer 2010). Insects are directly and/or indirectly an excellent nutritional option for humans because they are also part of the animal food webs in various land and water environments (DeFoliart 1999, Makkar et al. 2014, Kim et al. 2019). Edible insects are commercialized by national and international companies that employ quality-controlled manufacturing methods (Taylor 1975, Menzel and D’Aluisio 1998, DeFoliart 1999). It has even been extrapolated that by 2023, the global market of edible insects will surpass 522 million U.S, dollars (Han et al. 2017). In Mexico, anthropo-entomophagy is an ancient tradition (Ramos-Elorduy and Pino 1989). Much of the information on edible insect consumption and trade in Mexico has been obtained from direct observations made in the field, i.e., through surveys, questionnaires, and interviews conducted in general food markets, *tianguis* (a term derived from the Nahuatl word *tianquiztli* used to designate an open-air or on-the-road market), and restaurants, where diverse insect species for sale and consumption can be found (Mariaca 1994, Ramos-Elorduy et al. 2018).

Due to current demand, insects are the main ingredient in a variety of dishes (Govorushko 2017). Insects such as Oaxaca’s grasshoppers, the water bug called ‘axayacatl’, the waterfly pupae ‘poxi’, the bug eggs ‘ahuahutle’, and the fly larvae ‘cola de ratón’ are sold at retail in servings of <1 kg and wholesale in sacks of 50 kg in Oaxaca, Texcoco, Estado de México, Guanajuato, and Michoacán (Figueroa 2001, Pino et al. 2006, Ramos-Elorduy and Pino 2006, Ramos-Elorduy et al. 2007, Pino et al. 2016). Similarly, the ant larvae ‘escamoles’ are sold wholesale in Hidalgo and Estado de México (Ramos-Elorduy et al. 1998, Ramos-Elorduy and Pino 2001). The red maguey worm *Comadia redtembacheri* Hammerschmidt, 1848 (Lepidoptera:Cossidae) is among the most sought-after edible insects in rural areas, markets, *tianguis* and restaurants; this insect species is even used during the distillation of mezcal exported to the United States. In addition, Hemiptera–Heteroptera from the family Dactylopiidae ‘grana cochinilla’ (*Dactylopius coccus* Costa, 1835) is prized as a source of carminic acid and has multiple applications in food, sweets, soda, milk products, and pharmaceutical industries (Ramos-Elorduy et al. 2018).

Due to the high acceptance of entomophagy (insect consumption) in Mexico, several companies that produce and commercialize diverse foodstuffs prepared from insects, both for humans and as feed for farmed animals, do business in the states of Hidalgo, México, Morelos, Oaxaca, Puebla, and Querétaro (Pino et al. 2016).

In the state of Morelos, there is a long history of human consumption of a great variety of insects including grasshoppers, crickets, bugs ‘jumiles’, cicadas, ‘rotting wood worms’ and nopal worms, Saturnidae larvae ‘cuetlas’, red and white maguey worms, wasps, bees, and ants (Pino et al. 2015, Reyes-Prado et al. 2015, Reyes-Prado et al. 2016, Reyes-Prado and Pino 2020). Nevertheless, very few studies are available about their commercialization exist (Pino et al. 2016). In this context, we conceived the present study to investigate the edible insect species commercially exploited in the state of Morelos, including their prices, with an emphasis on the assessment of their economic importance in the life of the local people involved in this trade.

## Materials and Methods

### Study Area

**Fig. 1.**
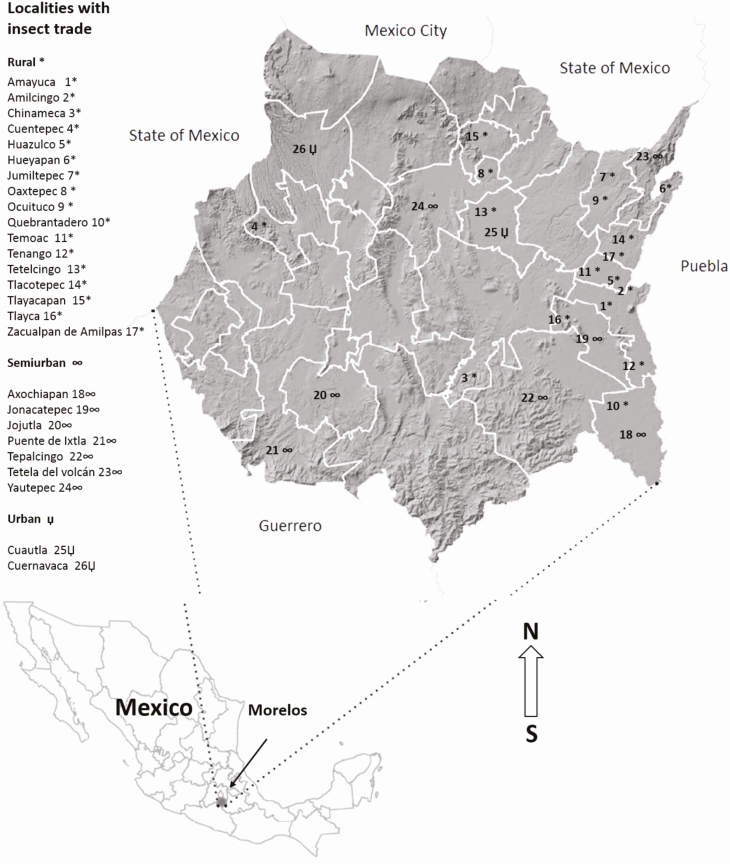
The localities (26) in the state of Morelos, Mexico, in which trade of edible insects was documented in preliminary visits to food markets.

The present study was carried out along the state of Morelos, which is located on the central part of Mexico, in the southern slope of the Cordillera del Ajusco and at the basin of the Balsas river, between the parallels 18°22′05″ and 19°07′10″N, and the meridians 93°37′08″ and 99°30′08″W (INEGI, 2019). Localities for sampling of the edible insects that are commercialized were selected based on preliminary visits aimed at finding out if there were an insect trade in the food markets, along with the social characteristics and indigenous groups present in the localities. The majority of people interviewed spoke two languages, an indigenous dialect (Nahuatl, Zapoteco, Tlapaneco, Mixteco, or Otomí) and Spanish. Interviews were carried out using questionnaires composed of open questions to obtain information of the economic and social context. Surveyed localities corresponded to villages and cities, which were divided into rural (0–10,000 inhabitants), semiurban (10,000 to 50,000 inhabitants), and urban (more than 50,000 inhabitants) considering the number of inhabitants and the services offered to the population. Insect trade was documented in 26 localities in our preliminary visits to food markets (Fig. 1).

### Collection of Edible Insects

Surveys were carried out at the food markets and *tianguis* of the selected localities in the state of Morelos, Mexico, during the four seasons of the years 2018 and 2019. *Tianguis* are provisional markets where people from neighboring villages gather to buy or sell food and household products. We recorded the following information from the insect samples bought in *tianguis* and/or food markets and those reported by the interviewed insect vendors: locality, common name, developmental stage commercialized, months of the year during which insects are sold, the container (or serving) in which they are displayed or sold, and their price.

In addition, insect samples were weighed (AccurisPrecision Balance W3200-120, Benchmark Scientific, Sayreville, NJ) at the Laboratory of Ecological Chemistry of the Autonomous University of The State of Morelos, Mexico. The edible insects were identified at the order level using taxonomic keys and references of the insect groups collected (Márquez 1962, Duzee 1917, Brown 1975, Beutelspacher 1980, Rolston et al. 1980, Rolston and MacDonald 1984, Beutelspacher and Balcázar 1994, Thomas 2000, Ortega and Thomas 2004, Triplehorn and Johnson 2005). Taxonomic identifications were later verified or corrected by experts from the Institute of Biology at the National Autonomous University of Mexico.

## Results and Discussion

In this study, we report the insects that are commercialized at the food markets or *tianguis* in the state of Morelos. The documented insects belong to the following orders: Orthoptera (two species), Hemiptera-Heteroptera (four species), and Lepidoptera (two species). We also report the families, scientific names, common names, edible developmental stages, marketing season, localities, containers in which they are exhibited and sold, weight of the insect servings, and the prices of each species (Table 1).

**Table 1.** Taxonomic relationships of edible insects, collected in food markets, and *tianguis* in various localities in the state of Morelos, Mexico

Order, Family, Scientific Name	Common name / Stage of edible development marketed/ Time of Sale	Locality (ies)	Container-insect weight in grams (Mean ± SEM*)	Price (mexican peso; 1 USD ≈ 20 MXN at the time of this study)
ORTHOPTERA Pyrgomorphidae *Sphenarium magnum* Márquez, 1962 *Sphenarium* sp.	Grasshoppers / Adults and nymphs / June–Nov.	Amayuca, Amilcingo, Cuentepec, Huazulco, Hueyapan, Tenango, Temoac Tetelcingo, Tlayca,	Cazuela Clay pots (56.6 ± 0.122)	10 MXN
			A Sardine can (83.2 ± 0.251)	20 MXN
		Jonacatepec, Ocuituco, Tlacotepec Tlayacapan, Tetela del Volcán, Quebrantadero, Zacualpan de Amilpas	‘Cazuela’ Clay pots (56.6 ± 0.122g)	10 MXN
			A Tuna can (74.7 ± 0.275)	15 MXN
			A Sardine Can (83.2 ± 0.251)	25 MXN
		Axochiapan, Cuautla, Cuernavaca, Jojutla, Oaxtepec Puente de Ixtla, Tepalcingo, Yautepec	‘Cazuela’ earthen (12 ± 0.316)	5 MXN
			‘Cazuela’ Clay pots (56.6 0.122)	10 MXN
			A Tuna can (74.7 ± 0.275)	15 MXN
			A Sardine Can (83.2 ± 0.251)	25 MXN o 30 MXN
HEMIPTERA-HETEROPTERA Pentatomidae *Euschistus egglestoni* Rolston, 1974 *Euschistus comptus* Walker, 1868 *Edessa* cordifera Walker, 1868 *Edessa conspersa* Stål, 1872	‘Jumiles’-‘chumiles’/ Adults and nymphs / Oct.–Feb.	Amayuca, Amilcingo, Chinameca., Huazulco, Jumiltepec, Ocuituco, Zacualpan de Amilpas, San Juan Chinameca.	Paper Cone (cucurucho; 15 ± 1.826)	5 MXN
			Paper Cone (26 ± 2.145)	10 MXN
			Plastic Bag (39 ± 6.745)	15 MXN
		Cuautla, Cuernavaca, Jojutla, Puente de Ixtla, Yautepec	Paper Cone (15 ± 1.826)	10 MXN
			Paper Cone (26 ± 2.145)	15 MXN
			Plastic Bag (39 ± 6.745)	20 MXN
LEPIDOPTERA Saturniidae *Arsenura armida* Cramer, 1979	‘Cuetlas’/ Larvae / June–Aug.	Zacualpan de Amilpas	A Sardine Can (88.5)	20 MXN
Cossidae *Comadia redtembacheri* Hammerschmidt, 1848	Maguey red worm / Larvae / Aug.–Nov.	Axochiapan, Quebrantadero	Plastic bag with 12 caterpillars (larvae) 28.2†	30 MXN

*SEM = standard error of mean.

^†^Only one sample was obtained.

The average weight of insect servings sold in each type of container was obtained. Even though insect samples were not purchased in all the surveyed localities, insects were exhibited and sold in similar containers and servings in all of them. Twenty-nine samples of grasshoppers were obtained, average weight of grasshopper servings for all the types of containers showed little variation as shown by the standard error (Table 1).

In the case of ‘jumiles’, 17 samples from different containers were acquired, a high variation in the standard error in the average weight of the sample from each container was observed (Table 1). This may be due to the fact that paper cones are made by hand and thus, the volume of the paper cones may vary, as well as the fact that ‘jumiles’ are sold alive and hence they can escape from the paper cone at the time of sale.

Lepidoptera such as cuetlas and red maguey worms are rarely sold in food markets and only one sample was obtained for each edible insect.

In the food markets, there were on average two to seven insect vendors who offered only grasshoppers, while others sold grasshoppers in addition to other products such as plums and boiled peanuts, among other foodstuffs. The great majority of vendors collected the insects from the field themselves either for personal consumption or for sale, but there were also resellers (people who buy the insects from the collector at a lower price in order to sell them at a higher price).

For their consumption, grasshoppers must be initially purged for 24 h, to avoid a bitter taste; afterward, they are placed in boiling salty water until they turn red in color. Then they are immediately roasted in a clay comal or cazuela (a circular and shallow metal or clay bowl). They can be spiced with a garlic sauce or fried only with salt and lemon. Grasshopper trade begins in June when the nymphs emerge and reaches its peak between July and October, when the insects reach a bigger size and are abundant.

**Fig. 2.**
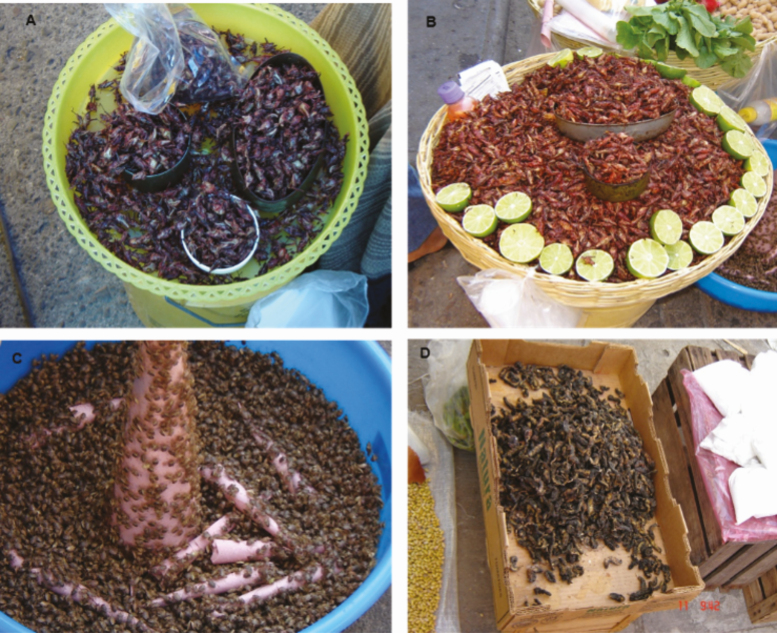
(A and B) Containers in which grasshoppers are sold. (C) Sale of *Euschistus sp*. ‘jumiles’ in ‘cucuruchos’ (paper cones). (D) Sale of ‘cuetlas’ *Arsenura armida* displayed in a cardboard box.

Adult grasshoppers are preferred by elderly people, who consider their flavor more pleasant than the taste of the nymphs. *Sphenarium* sp. and *Sphenarium magnum* Márquez, 1962 (Orthoptera: Acrididae) are traded in containers or servings of diverse sizes such as small clay pots or in tuna or sardine cans (Fig. 2A and B), they are also displayed in larger bowls or sacks holding10 kg, and their price varies according to the weight in each container.

**Fig. 3.**
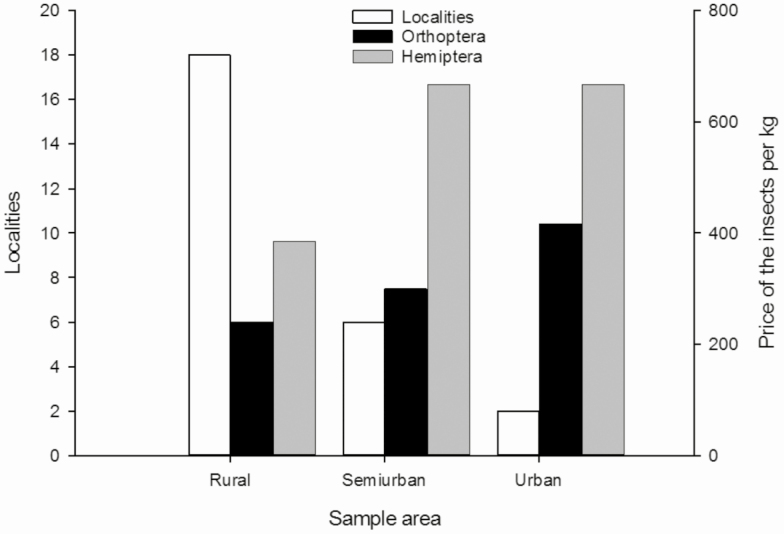
Localities and prices of the main insects that are commercialized in rural, semiurban, and urban regions.

In rural localities, where more indigenous people can be found, 240 MXN (Mexican peso; 1 USD ≈ 20 MXN at the time of this study) is the average price of grasshoppers per kilogram. A family of three to four members in an average of 4 h of work per day can collect for self-consumption or sale 1 kg of grasshoppers, while a single person can spend twice as long picking up the same amount. In semiurban localities, the price per kilogram reaches up to 300 MXN and in urban localities the cost rises to 417 MXN (Fig. 3). The price of grasshoppers in the semiurban localities of the state of Morelos is similar to that reported for the state of Oaxaca, which is 318 MXN per kilogram (Hernández et al. 2020). If we estimate that a person or a family collects a kilogram per day, each month they will have an income between 7,200 MXN and 12,510 MXN, which is a significant amount of money as the collector invests very little time doing this activity. Grasshoppers are sold on the food market 2 d after collection considering the amount of time invested for collection and preparation for consumption (purging of the insects for 24 h and spicing). However, vendors mentioned that grasshoppers can be preserved in the freezer or refrigerator for up to 6 mo, and be spiced again for consumption or sale when needed. The intermediary group that buys the insects at a cheap price from the collectors and sells them at a higher price in the cities gets a comparatively higher income. For example, in big cities like Cuautla and Cuernavaca, small servings of 12 may cost up to 5 MXN for city inhabitants who only want to try insects or are curious about their taste, thus by selling 10 to 25 servings, one can make an easy profit of 50 to 125 MXN (Table 1).

It is important to point out that even in some localities in the state of Morelos characterized by their high urbanization and industrial development, such as the capital city of Cuernavaca, there is a trend towards consumption and commercialization of grasshoppers. Pino et al. (2016) mentioned that these insects have a high market demand and therefore their consumption is accepted, and this has a significant socioeconomic impact both for the inhabitants of rural areas and for the people in the urban localities.

The ‘jumiles’ or ‘chumiles’ are exhibited alive in a bowl and are sold in ‘cucuruchos’ (paper cones; Fig. 2C). One can find up to three vendors that exclusively sell ‘jumiles’ on *tianguis* days. The price charged to consumers for these insects varies according to the locality: in small villages the cost of the ‘jumiles’ is lower than that in the bigger cities (Table 1). The price of a kilogram can even surpass 600 MXN (Fig. 3). ‘Jumiles’ from Taxco, Guerrero are commercially distributed at the intermunicipal and interstate level, and may be sold in certain localities of Morelos such as Jojutla. Lepidopteran caterpillars, like the red maguey worm, are commercially distributed at a smaller scale than grasshoppers and ‘jumiles’. They are sold alive or toasted with salt and accompanied by tomato sauce in bags with 12 larvae for 30 MXN. These caterpillars were documented in only two sites where they are exclusively sold at the Sunday *tianguis*, i.e., in Axochiapan and Quebrantadero. Other lepidopteran insects, like the larvae of *A. armida* known as ‘cuetlas’, are sold as prepared dishes or boiled in salted water on *tianguis* day in Zacualpan de Amilpas (Fig. 2D). The cost per serving (a sardine can) is 20 MXN, so if ten servings are sold, vendors may earn up to 200 MXN (Table 1).

The commercialization of many insect species is carried out during the time of their greatest abundance, generally during the rainy season. In certain zones of the state of Morelos, insect sales enable some vendors to obtain additional income to supplement their salary. The rural inhabitants with informal work (without a fixed salary such as agricultural workers, farmers, laborers, vegetable merchants, among others) have an income of between 100 and 150 MXN per day, in semiurban areas it varies between 100 and 180 MXN per day, and in urban areas it can reach up to 150–200 MXN per day. Inhabitants who have a formal job (workers) earn a minimum wage of 123 MXN per day in the rural area (CONASAMI 2020) and up to two times the minimum wage in urban locality. Therefore, the trade of insects allows insect sellers to obtain an income in addition to their salary. However, this may turn out to be insufficient considering the higher levels of unemployment among this population. Some resellers, sell the edible insects at a higher price than collectors do. In several cases the resellers make more profit than the collectors.

For the largest number of vendors of the state of Morelos, the collection and sale of insects represents a support for their income and welfare economy at the time of peak trade; in addition, this natural resource is wholly free for the collectors and the job only involves the collection labor, an activity that is sometimes done by entire families. In the state of Puebla, for example, 50 tons of grasshoppers are harvested and sold annually with a value of 2.5 million MXN. This means that it is a significant economic activity for the people in rural areas who live in a subsistence economy with low paid temporary jobs (Figueroa 2001).

Entomophagy ‘could’ be a solution for food security; nevertheless, with rising insect consumption, the level of commercialization will increase. Hence, it is necessary to devise regulations for the breeding, production, distribution, and commercialization of edible insects not only in Mexico, but also in other developing countries that are promising good business opportunities (Baiano 2020, Imathiu 2020). In several countries throughout the world, successful models exist to scale mass breeding of several insect species, such as *Hermetia illucens* Linnaeus, 1758 (Diptera: Stratiomyidae) (black soldier fly), *Musca domestica* Linnaeus, 1758 (Diptera: Muscidae) (domestic fly), *Tenebrio molitor* Linnaeus, 1758 (Coleoptera: Tenebrionidae) (yellow meal worm), and *Acheta domesticus* Linnaeus, 1758 (Orthoptera: Gryllidae) (domestic cricket; van Huis and Tomberlin 2017). These insects are being commercialized by insect-producing industries like AgriProtein in South Africa and Enviroflight in the United States, which exemplify the present and future economic potential of insect production industries worldwide (van Huis et al. 2013, Han et al. 2017).

Edible insects are a natural resource and it is necessary to formulate strategies to avoid their over-exploitation. For example, one could consider the sustainable management of insects like grasshoppers, which are crop pests, but widely used as food for humans or feed for animals, instead of exterminating them with harmful insecticides (Litton 1993, Ramos-Elorduy et al. 2006, Hernández et al. 2020).

From the present study, we could conclude that grasshoppers and ‘jumiles’ are the insects that are commercially exploited in most of the localities in the state of Morelos. The remaining species are a subject of trade to a lesser degree and in a fewer number of localities. These insects are collected mainly for self-consumption by marginal populations. Thus, it could be concluded that insects are a promising resource for the economy, especially in the state of Morelos, where the minimum wage is still insufficient to satisfy the needs of the local population.
